# Age‐Period‐Cohort Analysis of Diabetes Incidence and Mortality in China and Globally, 1990–2019

**DOI:** 10.1111/1753-0407.70051

**Published:** 2025-01-30

**Authors:** Jinli Liu, Mingwang Shen, Guihua Zhuang, Lei Zhang

**Affiliations:** ^1^ Med‐X Institute, Center for Immunological and Metabolic Diseases The First Affiliated Hospital of Xi'an Jiaotong University Xi'an Shaanxi China; ^2^ China‐Australia Joint Research Center for Infectious Diseases, School of Public Health Xi'an Jiaotong University Health Science Center Xi'an Shaanxi People's Republic of China; ^3^ Key Laboratory of Environment and Genes Related to Diseases (Xi'an Jiaotong University), Ministry of Education Xi'an China; ^4^ Melbourne Sexual Health Centre Alfred Health Melbourne Australia; ^5^ Central Clinical School, Faculty of Medicine Monash University Melbourne Australia

Diabetes has rapidly emerged as a critical global health emergency of the 21st century [1, 2]. China, bearing the highest global burden of diabetes, witnessed its prevalence rise from 10.6% in 2002 to 12.4% in 2018 [3, 4]. Liu et al. [5] reported a 0.89% annual increase in global diabetes incidence during 1990–2017. Diabetes mortality has remained stable in China, with an increase from 10.1/100 000 in 1990 to 10.3/100 000 in 2013 [6]. Diabetes risk factors are diverse, including genetic, environmental, and metabolic elements [7, 8], as well as family history [7], socioeconomic development [9], physical inactivity [10], overweight and obesity [11], and dietary changes [12, 13, 14, 15]. In recent years, these factors have evolved, shaping distinct temporal trends in diabetes‐related diseases and influencing age, period, and cohort trends in diabetes incidence and mortality in both China and globally.

Previous studies have focused on the overall changes in the disease burden of diabetes over time [16]. The present study aimed to explore the overall trends in diabetes incidence and mortality in China from 1990 to 2019, and then further analyze the age, period, and cohort‐specific trends, comparing these with corresponding trends across the three dimensions at the global level. This study collected data from the Global Burden of Disease Study 2019, which includes only type 1 and type 2 diabetes among individuals aged 20 years and older. A log‐linear model was used to analyze overall trends in diabetes incidence and mortality, followed by an age‐period cohort model to examine age, period, and cohort trends in both China and globally, with comparisons made between the two populations.

The study highlighted similar age‐related patterns in diabetes incidence risk in both China and globally, with an initial increase followed by a decline. However, the peak age for diabetes incidence risk in China was in the 50–54 age group, whereas in the global population, it occurred in the 55–59 age group (Figure 1a). China's peak age for diabetes incidence risk was approximately 5 years younger than the global peak age, consistent with previous findings [17]. Before 2010, diabetes incidence risk in China was higher than the global average, but after 2010, it fell below the global average (Figure 1b). This suggests that diabetes prevention and control measures in China have been notably effective since 2010, highlighting the success of preventive policies [18]. The diabetes incidence risk in individuals born in China between 1985 and 1999 has significantly increased compared to earlier cohorts and is also higher than that of the global population born in the same period (Figure 1c). This cohort likely experienced a sharp rise in diabetes risk due to China's rapid economic development and improved living standards during the period of economic reform and opening up [19, 20]. The age‐period‐cohort effects on diabetes mortality risk in China align closely with global trends. Mortality risk increased with age and as time progressed, while it decreased with later birth cohorts (Figure 1d–f). The study provides a deeper understanding of diabetes prevention, treatment, and management in China, laying a theoretical foundation for national diabetes control strategies and the achievement of the “Healthy China 2030” goals.

**FIGURE 1:**
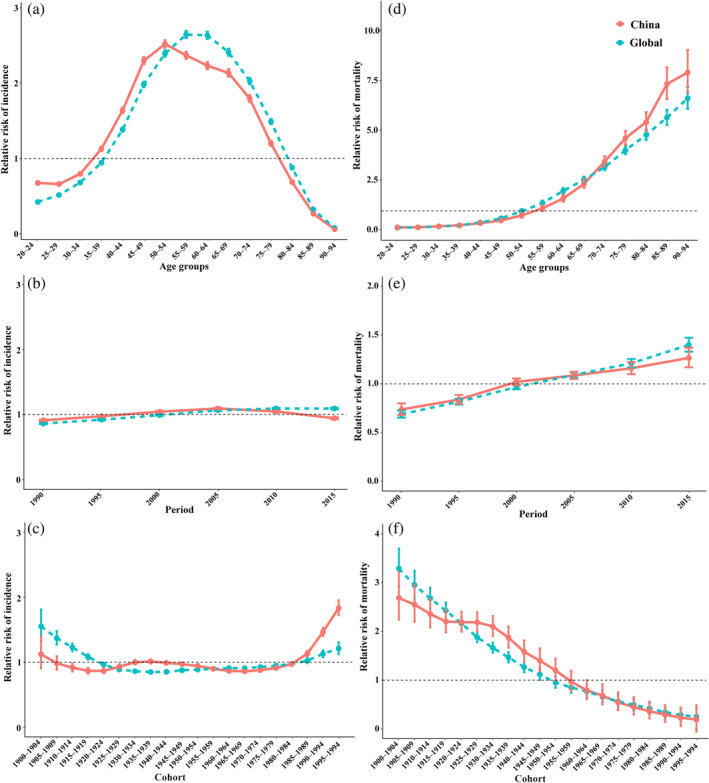
Diabetes incidence and mortality relative risks (RR) due to age (a, d), period (b, e), and cohort (c, f) trends in China and globally.

## Author Contributions

L.Z. and J.L. substantially contributed by developing the conceptual framework and design of the study. J.L. wrote the first draft of the manuscript and performed the statistical analysis. All authors contributed to the interpretation of the results and writing. L.Z., G.Z. and M.S. critically revised the manuscript for important intellectual content. All authors have approved the final version to be published. L.Z. is the guarantor of this work and, as such, had full access to all the data in the study and takes responsibility for the integrity of the data and the accuracy of the data analysis.

## Conflicts of Interest

The authors declare no conflicts of interest.

## Data Availability

The original contributions presented in the study are included in the article and online Supplementary Information. Data can be obtained at this website: http://ghdx.healthdata.org/gbd‐results‐tool. Further inquiries can be directed to the corresponding author.
